# A Review of the Recent Epidemiology of Zika Virus Infection

**DOI:** 10.4269/ajtmh.24-0420

**Published:** 2025-02-11

**Authors:** Ingrid B. Rabe, Susan L. Hills, Joana M. Haussig, Allison T. Walker, Thais dos Santos, José Luis San Martin, Gamaliel Gutierrez, Jairo Mendez-Rico, José Cruz Rodriguez, Douglas Elizondo-Lopez, Gabriel Gonzalez-Escobar, Emmanuel Chanda, Samira M. Al Eryani, Chiori Kodama, Aya Yajima, Manish Kakkar, Masaya Kato, Pushpa R. Wijesinghe, Sudath Samaraweera, Hannah Brindle, Hasitha Tissera, James Kelley, Eve Lackritz, Diana P. Rojas

**Affiliations:** ^1^ Department of Epidemic and Pandemic Preparedness and Prevention, World Health Organization, Geneva, Switzerland;; ^2^ Division of Vector-Borne Diseases, U.S. Centers for Disease Control and Prevention, Fort Collins, Colorado;; ^3^ European Centre for Disease Prevention and Control, Solna, Sweden;; ^4^ Divisions of Global Migration Health, U.S. Centers for Disease Control and Prevention, Atlanta, Georgia;; ^5^ World Health Organization Regional Office for the Americas/Pan American Health Organization, Washington, District of Columbia;; ^6^ WHO Regional Office for Africa, Brazzaville, Republic of the Congo;; ^7^ World Health Organization Regional Office for the Eastern Mediterranean, Cairo, Egypt;; ^8^ World Health Organization Regional Office for South-East Asia, New Delhi, India;; ^9^ World Health Organization Regional Office for the Western Pacific, Manila, Philippines;; ^10^ Center for Infectious Disease Research and Policy, Minneapolis, Minnesota

## Abstract

Zika virus (ZIKV) is a flavivirus transmitted primarily by the bite of infected *Aedes* species mosquitoes. Although typically asymptomatic or causing mild symptoms and infrequent neurological disease in older children and adults, infection during pregnancy can result in severe congenital malformations and neurodevelopmental deficits. We conducted a review of published literature and official data sources to describe recent Zika epidemiological trends, building on WHO updates posted in 2019 and 2022. Globally, cases declined after the height of ZIKV transmission in the Americas in 2015–2016; however, transmission continues across multiple regions, with intermittent outbreaks reported. As of December 2023, there is documented evidence of current or prior autochthonous mosquito-borne ZIKV transmission in 92 countries and territories; most recently, Guinea, Mali, and Sri Lanka were included on the basis of recent or retrospective testing of specimens collected during surveillance activities or studies. The abundance of asymptomatic and mild infections and limited diagnostic testing suggest that transmission in many locations likely remains underrecognized. Public health authorities, clinicians, communities at risk, and travelers should remain alert to the possibility of ZIKV transmission and implement measures to limit the risk of infection with ZIKV and other *Aedes*-borne arboviruses. To strengthen surveillance for ZIKV infections and congenital disease, targeted surveillance using clear case definitions and epidemiologically appropriate laboratory testing algorithms should be applied.

## INTRODUCTION

Zika virus (ZIKV) is a flavivirus transmitted primarily by the bite of infected *Aedes* (*Stegomyia* subspecies) mosquitoes. Zika virus is transmitted predominantly by *Aedes aegypti*, although *Aedes albopictus* mosquitoes are also competent vectors.^1^ In addition, ZIKV can be transmitted sexually, from mother to fetus during pregnancy or around the time of birth, through laboratory exposure, and probably through transfusion of infected blood products.^2^^–^^5^

Zika virus infection in older children and adults is typically asymptomatic or causes mild disease. It can, however, result in rare but severe outcomes, including Guillain-Barré syndrome, transverse myelitis, and other systemic and neurologic sequelae.^6^ Infection during pregnancy can cause severe adverse outcomes that include increased risk of preterm birth, fetal death and stillbirth, and congenital malformations collectively characterized in their most severe form as congenital Zika syndrome (CZS).^7^ Clinical components of CZS include microcephaly and other abnormal cranial morphologies, abnormal brain development, limb contractures, eye abnormalities, brain calcifications, and other neurologic clinical features; the quantitative risk of these outcomes remains under investigation.^8^ Recognition of the severe congenital malformations associated with ZIKV infection in the Americas, and retrospective confirmation of similar malformations in French Polynesia, prompted the Director General of the WHO to declare a public health emergency of international concern (PHEIC) in February of 2016.^9^ Studies are ongoing to quantify the association between congenital infection and milder sequelae that may not be immediately recognized at birth, including developmental delay and learning disabilities.^10^^–^^12^

Genetic sequencing of ZIKV isolates and ZIKV reverse transcription polymerase chain reaction (RT-PCR) RNA products has shed some light on patterns of transmission and global spread.^13^^,^^14^ ZIKVs are categorized phylogenetically into African and Asian lineages. The ZIKV African lineage has been isolated sporadically in nonhuman and human specimens since 1947.^15^ Asian lineage viruses were first isolated in Malaysia in 1966, followed by isolations in other Asian countries; ZIKVs of the same lineage were isolated in the Pacific Islands from 2007 onwards.^16^^,^^17^ A descendant of the Asian lineage now commonly referred to as the American sublineage caused the 2015–2016 epidemic in the Americas.^18^ More recently, ZIKV African lineage genomic sequences were identified in Brazil through secondary analysis of RNA sequences in specimens of mosquito and nonhuman primate origin.^19^ The effect of introduction of ZIKV lineages or sublineages into areas where others have previously circulated is not known at this time, and differences in epidemic potential and pathogenicity remain under investigation. Congenital microcephaly following maternal ZIKV infection during pregnancy has been documented in both ancestral and American strains of the ZIKV Asian lineage.^20^ In contrast, to date, adverse pregnancy outcomes and cases of CZS caused by ZIKV African lineage viruses have not been recognized, and it is not known whether this is because they do not occur or because of limitations of detection and surveillance. Studies of the African lineage in vitro and in animal models demonstrated increased pathogenesis in pregnancy compared with that demonstrated by the Asian lineage, suggesting a propensity to cause fetal loss rather than birth defects.^21^

The WHO has published data on cases since Zika was first declared a PHEIC, initially those reported to the organization under International Health Regulations and, following termination of the PHEIC, based on the review of literature and official reports of Zika cases and clusters.^22^^,^^23^ The most recent update was posted in February 2022, summarizing epidemiological data published by regions and member states through December 31, 2021.^24^

During the PHEIC in 2016, an expert panel was convened that included representation from WHO Headquarters, the WHO Regional Office for the Americas (AMRO)/Pan American Health Organization (PAHO), the European Centre for Disease Prevention and Control, and the U.S. Centers for Disease Control and Prevention (U.S. CDC) to evaluate the existing evidence of current or prior transmission of ZIKV across countries and territories globally. This informed the construction of a country classification scheme to allow an assessment of the possibility of infection for residents of, and travelers to, countries with ZIKV transmission.^22^ Since 2017, following the peak of ZIKV transmission in the Americas, Zika epidemiology has changed dramatically. Global reported case numbers have declined substantially, but sporadic cases and occasional outbreaks are still being reported. Areas without previously recognized transmission continue to be identified, often from retrospective testing of patient samples and though testing of infected travelers returning from these areas. The aim of the study was to review and contextualize recent data on Zika epidemiology.

## MATERIALS AND METHODS

We conducted a literature review and investigated additional official data sources to describe the global status of Zika and changes in geographic distribution of its key mosquito vector, *Ae. aegypti*, as of December 31, 2023. We focused specifically on data indicating changes in the epidemiologic situation since the last WHO Zika Epidemiological Update in 2022 on the basis of detection of new transmission events or retrospective investigations elucidating unrecognized areas of confirmed transmission.

### Review of published literature and official data sources.

Using the baseline of the database compiled by the multiagency epidemiology team during the PHEIC, we conducted an iterative PubMed literature review of publications from December 2021 (at the end of the literature review period for the most recent WHO Zika Epidemiological Update) through December 2023 with the search terms “Zika” and “Zika virus” individually and in combination with “distribution,” “epidemiology,” and “detection,” respectively. A similar search was conducted with the term “*Aedes aegypti.*” We reviewed Ministry of Health (MoH) websites of countries with established *Ae. aegypti* populations to identify case reports. We also reviewed GenBank ZIKV sequence submissions to determine where specimens were collected and whether there was sufficient indication of where exposure had occurred. For aggregate surveillance totals, the definition of a Zika case was that specified in the case definition applied in the respective reporting country. Regional definitions used by the WHO AMRO/PAHO are available online.^25^

### Regional office communications with member states.

WHO regional emergencies and neglected tropical disease technical/medical officers and WHO arbovirus consultants communicated with member states to identify or confirm reports of autochthonous ZIKV transmission that were not captured in the literature review or officially published data sources. They also confirmed *Ae. aegypti* presence when countries with a newly identified presence of this vector were identified.

### Definition of current or previous transmission.

In accordance with the definition used by the expert panel during the PHEIC, current or previous ZIKV transmission in a country or territory was defined as 1) the occurrence of a laboratory-confirmed autochthonous, mosquito-borne case of ZIKV infection, whether it was detected and reported by the country/territory where infection occurred or by another country by diagnosis of a returning traveler, or 2) laboratory-confirmed infection in an animal or mosquito vector.^16^ Laboratory confirmation was defined as 1) detection of the virus or viral RNA in humans, mosquitoes, or other animals by isolation or nucleic acid amplification testing or 2) serologic confirmation in humans with gold standard neutralizing antibody tests with testing for all appropriate potentially cross-reactive flaviviruses that might be circulating in the area of interest. Because of testing and interpretation limitations with serological data predating 1980, these data were not included as evidence of transmission.

## RESULTS

Following the termination of the PHEIC in November 2016, reported Zika cases have declined, but circulation persists at low levels in some countries in the Americas, Southeast Asia, the Western Pacific, and Africa.^23^^,^^24^^,^^26^^,^^27^ At the same time, few countries currently implement systematic ZIKV laboratory testing and surveillance to the extent that this was done during the PHEIC.^25^^,^^28^ Since the last WHO Zika Epidemiological Update posted in February of 2022, which covered the period up to December 31, 2021, additional data on Zika epidemiology have been reported, and ongoing transmission, clusters, or outbreaks have been detected in various locations.^24^ The Region of the Americas maintains ongoing *Aedes*-borne arbovirus surveillance and has continued to report the most cases globally.^29^ Outside of the Americas, clusters of Zika cases were reported from India (2022 and 2023), Singapore (2023), and Thailand (2023), and sporadic cases of disease were reported in travelers returning from Thailand, the Maldives, and India.^30^^–^^32^ Additional reports providing evidence of ZIKV infection in countries without previous confirmation of autochthonous ZIKV transmission included confirmation of infection from retrospective testing of patient sera in Sri Lanka, testing in a pregnant woman residing in Guinea, and RT-PCR confirmation of infection in a cluster of ZIKV infections in Mali.^33^^,^^34^

Globally, as of December 2023, there has been documented evidence of autochthonous mosquito-borne transmission of ZIKV in 92 countries and territories (Table 1).^35^ These countries and territories are distributed across five of the six WHO regions, the exception being the Eastern Mediterranean Region, where autochthonous ZIKV transmission has not been reported but transmission of other *Aedes*-borne arboviruses is known to occur.^36^ In total, 60 countries and territories have evidence of established *Ae. aegypti* vector populations but have not yet documented autochthonous ZIKV transmission (Table 2). This includes most recently the addition of Mauritania to the WHO list, based on literature that was missed in previous WHO updates, and Cyprus, where the vector was recently detected.^37^^,^^38^ More detailed information follows on changes and updates in ZIKV epidemiology in the six WHO regions as documented since January 2022.

**Table 1:** Countries and territories with current or previous ZIKV transmission,* by WHO region, December 2023

WHO Region	Country/Territory	Total No. of Countries
African Region	Angola, Burkina Faso, Burundi, Cabo Verde, Cameroon, Central African Republic, Côte d’Ivoire, Ethiopia, Gabon, Guinea, Guinea-Bissau, Kenya, Mali, Nigeria, Senegal, Uganda	16
Region of the Americas	Anguilla, Antigua and Barbuda, Argentina, Aruba, Bahamas, Barbados, Belize, Bolivia (Plurinational State of), Bonaire, Sint Eustatius and Saba, Brazil, British Virgin Islands, Cayman Islands, Colombia, Costa Rica, Cuba, Curaçao, Dominica, Dominican Republic, Ecuador, El Salvador, French Guiana, Grenada, Guadeloupe, Guatemala, Guyana, Haiti, Honduras, Easter Island– Chile, Jamaica, Martinique, Mexico, Montserrat, Nicaragua, Panama, Paraguay, Peru, Puerto Rico, Saint Barthélemy, Saint Kitts and Nevis, Saint Lucia, Saint Martin, Saint Vincent and the Grenadines, Saint Maarten, Suriname, Trinidad and Tobago, Turks and Caicos, United States of America, United States Virgin Islands, Venezuela (Bolivarian Republic of)	49
South-East Asia Region	Bangladesh, India, Indonesia, Maldives, Myanmar, Sri Lanka, Thailand	7
Western Pacific Region	American Samoa, Cambodia, Cook Islands, Fiji, French Polynesia, Lao People’s Democratic Republic, Marshall Islands, Malaysia, Micronesia (Federated States of), New Caledonia, Palau, Papua New Guinea, Philippines, Samoa, Singapore, Solomon Islands, Tonga, Vanuatu, Vietnam	19
European Region	France (Var department)	1
Total	–	92

ZIKV = Zika virus.

* A laboratory-confirmed autochthonous, vector-borne case of ZIKV infection in a country or territory, whether it is detected and reported by the country/territory where infection occurred or by another country by diagnosis of a returning traveler. Autochthonous infection is considered an infection acquired in-country, i.e., among patients with no history of travel during the incubation period or who have traveled exclusively to nonaffected areas during the incubation period. Evidence of autochthonous, mosquito-borne transmission includes those countries with known historical laboratory evidence of ZIKV circulation based on published, peer-reviewed literature as well as all ZIKV surveillance data whether detected and reported by the country where infection occurred or by another country reporting a confirmed case in a returning traveler. Laboratory criteria to ascertain the presence of ZIKV in past studies are as follows: 1) detection of the virus in humans, mosquitoes, or animals; 2) serologic confirmation of ZIKV infection with tests conducted after 1980 and considered as confirmed infection on expert review based on testing for all appropriate cross-reactive flaviviruses and utilization of comprehensive testing methodologies. Because of testing and interpretation limitations with serological data antedating 1980, these data were not included as evidence of transmission.

**Table 2:** Countries and territories with established *Aedes aegypti* mosquito vectors, but no known cases of Zika virus transmission, by WHO region, December 2023

WHO Region	Country/Territory	Total No. of Countries
African Region	Benin, Botswana, Chad, Comoros, Congo, Democratic Republic of the Congo, Equatorial Guinea, Eritrea, Gambia, Ghana, Liberia, Madagascar, Malawi, Mauritania, Mauritius, Mayotte, Mozambique, Namibia, Niger, Réunion, Rwanda, Sao Tome and Principe, Seychelles, Sierra Leone, South Africa, South Sudan, Togo, United Republic of Tanzania, Zambia, Zimbabwe	30
Region of the Americas	Uruguay	1
Eastern Mediterranean Region	Afghanistan, Djibouti, Egypt, Oman, Pakistan, Saudi Arabia, Somalia, Sudan, Yemen	9
European Region	Cyprus, Georgia, Região Autónoma da Madeira – Portugal, Russian Federation, Turkey	5
South-East Asia Region	Bhutan, Nepal, Timor-Leste	3
Western Pacific Region	Australia, Brunei Darussalam, China, Christmas Island, Guam, Kiribati, Nauru, Niue, Northern Mariana Islands (Commonwealth of the), Tokelau, Tuvalu, Wallis and Futuna	12
Total	–	60

### African Region.

Although ZIKV was originally identified in Uganda in 1947, systematic surveillance data on current and prior transmission in the African Region are limited.^39^ Autochthonous transmission has now been documented in 16 countries; however, ZIKV is not reportable in 33 (70%) countries in the region.^40^ Information on the distribution of the original African viral lineages is sparse due to limited case detection and genomic sequencing capacity. More recently, broader differential diagnostic testing of specimens from suspected arboviral disease cases, including by multiplex RT-PCR, enabled detection of ZIKV infections in 2023 in Senegal, which had recorded prior transmission, and for the first time in Mali.^41^ The only evidence of microcephaly in the offspring of women in Africa followed detection (importation/emergence) of the Asian lineage ZIKV, first in Cabo Verde in 2015–2016 and subsequently in Angola in 2016–2017, in association with the heightened transmission in the Americas.^42^^–^^44^

Since the Zika PHEIC, evidence of ZIKV transmission has been sought in numerous countries in the African Region through research studies and special projects.^45^^–^^47^ Many of these studies were retrospective serosurveys that included ZIKV-specific neutralization testing but did not include cross-neutralization testing to exclude all potentially cross-reactive flaviviruses. Without active surveillance to detect either Zika cases or ZIKV-associated developmental defects, the capacity to detect transmission remains low, although increased testing of specimens by multiplex RT-PCR has resulted in detection of ZIKV infections.

Key data identified during the past 2 years pertaining to confirmed transmission and *Ae. aegypti* presence in the African Region include the following.

#### Guinea.

Researchers in Guinea conducted a study among febrile patients at four discrete timepoints from May 2018 to July 2021; patients’ blood specimens were tested for ZIKV RNA by RT-PCR and for IgM and IgG antibodies against ZIKV NS1 antigen.^33^ Blood collected in 2018 from a 27-year-old pregnant woman, at 16 weeks of gestation, 5 or 6 days after the onset of headache, fever, and weakness, tested positive for ZIKV RNA by RT-PCR testing, and IgM antibodies were detected by ELISA testing; IgG was not detected. She presented again at 20 days after illness onset, at which time RNA and IgG antibodies were detectable but IgM antibodies were no longer detectable. Virus was cultured from the initial specimen and sequenced (GenBank ID MN025403), identifying a ZIKV African lineage virus closely related to West African strains isolated from mosquito pools in Senegal. The woman had no documented travel history to areas of ZIKV transmission or potential sexual exposure to ZIKV listed, and the case was thus consistent with local acquisition of infection. The patient was lost to follow-up, and the outcome of the pregnancy was thus not documented. Overall, in the study, 116 specimens were collected and tested serologically for ZIKV IgM, and an average of 14.7% were seropositive per year; however, gold standard testing by neutralizing antibody testing was not performed, and cross-reactivity or nonspecific reactivity might have confounded results.

#### Mali.

In December 2023, the Mali MoH reported the first 12 RT-PCR-confirmed cases of Zika in the country.^41^ Further testing as of December 24, 2023 confirmed a total of 22 cases from 10 health districts in Koulikoro region (9), Sikasso region (1), and Bamako district (12). No deaths or information on pregnancy or adverse outcomes have been reported.

#### Nigeria.

Although Nigeria was the first country in which human cases of Zika were documented, only sporadic cases have been reported since.^48^ A study of pregnant women in northwest Nigeria in 2022 found that six women had detectable RNA on RT-PCR testing, constituting the most recent molecular evidence of autochthonous ZIKV transmission in the country.^49^

#### Senegal.

In week 49 of 2023, Senegal health authorities reported the confirmation of two Zika cases, in one female and one 18-year-old male, from the districts of Sédhiou and Sokone, respectively, through RT-PCR testing.^41^^,^^50^ Data on the female patient’s age and pregnancy status were not available.

#### Mauritania.

Evidence of *Ae. aegypti* was retrospectively identified from previous publications. Mauritania is now represented in WHO tables and maps as a country with *Ae. aegypti* but without documented evidence of ZIKV transmission.^37^^,^^39^^,^^51^^,^^52^

### Region of the Americas.

AMRO/PAHO maintains data on reported cases of Zika, and data are publicly available through the Public Health Information Platform for the Americas.^25^ Data from ongoing surveillance are reported by countries and territories directly to PAHO/WHO or collected from epidemiological bulletins posted on MoH websites. A summary of reported case numbers by country and subregion and a Zika epidemiologic summary are updated systematically and posted on the PAHO website.^29^ Country case numbers are not directly comparable, however, because some countries report only laboratory-confirmed cases and others report clinically suspected Zika cases that typically lack laboratory confirmation. Bermuda, Canada, mainland Chile, and Uruguay have never reported autochthonous, vector-borne transmission of ZIKV.

Incidence of ZIKV infection peaked in the Americas in 2016 and declined substantially thereafter. Zika cases continue to be reported in 14 to 15 countries annually, and the Americas remains the region with the highest number of yearly reported Zika cases. After 2016, the total number of reported cases per annum fluctuated, ranging from 22,978 in 2019 to 56,904 in 2017.^25^ Preliminary data from 2023, a year with increased dengue virus (DENV) and chikungunya virus transmission in the Americas, show 55,813 Zika cases (11% laboratory confirmed) reported from 14 countries, including four deaths, and 97% of the cases were reported from Brazil.^53^^,^^54^ Some reporting jurisdictions, particularly relatively smaller islands and territories, have reported no Zika cases since 2017. However, while some have maintained strong surveillance programs that indicate that transmission is likely interrupted, surveillance and reporting are not uniform and might not be sufficiently sensitive to detect low levels of transmission. In the period 2018–2022, Zika cases reported in the United States declined from 222 to 22; no autochthonous transmission has been reported in the continental United States since 2018, and no cases reported from U.S. territories since 2019 have met laboratory criteria for case confirmation.^55^

Data on the countries reporting most of the recent Zika cases in the Region of the Americas include the following.

#### Brazil.

Brazil was the country that first detected ZIKV in the continental Americas, in April 2015. As the most populous and largest country in South America, with ecological conditions favorable to *Aedes*-borne arbovirus transmission and a strong epidemiological surveillance system, Brazil remains the country with the highest reported case numbers globally. Total reported cases peaked in 2016 at 273,904 for a cumulative incidence of 131 cases per 100,000 population; 128,793 (47%) of cases were laboratory confirmed.^25^ Although case numbers declined from 2017 onwards, over 100,000 cases in total have been reported from Brazil since then.^56^ Annual cumulative case numbers from 2017 to 2022 ranged from a minimum of 17,496 (3,617; 21% laboratory confirmed) in 2021 to 34,176 (3,238; 9% laboratory confirmed) in 2022.^25^ In 2023, a total of 54,116 cases were reported for a cumulative incidence of 25 per 100,000 population; 6,201 (11%) of cases were laboratory confirmed. An analysis of cases reported at the municipal level from 2017–2020 showed that most were reported from northeastern Brazil, although hotspots were identified in all five regions, and cases clustered temporally in the first 6 months of the year.^56^

#### Bolivia.

Bolivia has reported variable case numbers annually, but in 2023, more cases were reported than in any of the preceding 5 years, with 881 cases for a cumulative incidence of 7.45 per 100,000 population, compared with 293 cases in 2019, 728 in 2020, 125 in 2021, and 190 in 2022. However, only seven of the cases reported in 2023 were laboratory confirmed.^25^

### Eastern Mediterranean Region.

Autochthonous ZIKV transmission has not yet been definitively reported in the Eastern Mediterranean Region, which spans parts of northern Africa and the Middle East, but transmission of other *Aedes*-borne arboviruses is known to occur.^36^^,^^57^ A single case of ZIKV infection based on serology was reported in Sudan, but the testing described and gaps in travel history meant that the case did not meet the definition used in this review of confirmed current or past transmission.^58^ The presence of *Ae. aegypti* in nine of the countries, *Ae. albopictus* recorded in several others, increased reports of dengue outbreaks annually, and the increased risk of arbovirus transmission among mobile and displaced populations have prompted increased efforts to identify vector introduction in the region and to enhance surveillance for ZIKV and other *Aedes*-borne arboviral diseases.^57^^,^^59^^–^^64^

### European Region.

In the European Region, cases in travelers returning from areas of endemicity have been reported since 2015 but have remained at low levels (<70 imported cases per year) since 2018. However, the first and only autochthonous mosquito-borne transmission recorded to date was in France in 2019, where ZIKV infections were laboratory confirmed in three people with no travel or sexual exposure history.^65^ Vector surveillance remains a priority within the region, with expanded geographic detection of *Ae. albopictus* and establishment of *Ae. aegypti* in Cyprus.^38^^,^^66^ In addition, recent increases in autochthonous dengue cases in France, Italy, and Spain have raised concerns about the risk of transmission of other *Aedes*-borne arboviruses, including ZIKV.^67^

### South-East Asia Region.

ZIKV has been circulating since at least the 1960s, with sporadic cases and small outbreaks reported in several countries of the South-East Asia Region since then. The region remains at substantial risk for ZIKV transmission because of the presence of competent vectors, often in high densities, and highly populated urban centers where infected humans serve as the source for ongoing transmission. Key data on transmission in the region over the past 2 years include the following.

#### India.

During the past several years, India has reported occasional outbreaks of Zika, including in 2018 in Jaipur, Rajasthan, and Madhya Pradesh and in 2021 in Kerala in May–July and Maharashtra in July.^23^^,^^24^ Among specimens collected from patients with possible arboviral disease and tested at sentinel surveillance sites across India from May to October 2021, 67 were found to have detectable ZIKV RNA, 60 of which were collected in the outbreak area in Kerala and the remaining seven in six states in India outside of outbreak areas; however, it is not clear that travel was ruled out in the source patients.^68^ In late 2021, an outbreak was reported in Kanpur, Uttar Pradesh. The first confirmed identified case was in a 56-year-old male who presented with less common clinical features of acute respiratory distress syndrome and multiorgan failure.^69^ Infections were subsequently confirmed in 126 people in Kanpur and adjoining areas, identified through active surveillance that focused on neighborhood contacts, pregnant women, and people with compatible symptoms; however, most of the people with confirmed infections were asymptomatic.^70^ Among the cases, ZIKV infection was confirmed by RT-PCR in a symptomatic woman in her first trimester of pregnancy. Sequential ultrasound follow-up was conducted, and at 32 weeks gestation, fetal radiological stigmata consistent with CZS were detected; the pregnancy ended in intrauterine demise at 34 weeks gestation.^71^ There was a single case of ZIKV reported in Raichur district, Karnataka, in 2022 and several sporadic cases and clusters identified in 2023, including a cluster of eight cases in Kerala and three cases in Kohapur district, Maharashtra.^72^^,^^73^

#### Maldives.

In May 2023, two travelers returning from Maldives to China were reported as having confirmed ZIKV infections.^32^ Maldives was already included in the list of countries with autochthonous transmission based on confirmed infections in travelers from 2015 onwards. Three cases were detected in-country during active surveillance in 2016–2017, but systematic testing has not been routinely performed since 2018.^28^

#### Sri Lanka.

In a retrospective study published in 2023 of patients suspected of having DENV infections from 2017–2019, researchers detected ZIKV RNA in specimens from six patients who presented to two hospitals in the central and western regions of the country.^34^ In addition, IgM and IgG ELISA testing with focus reduction cross-neutralization testing for confirmation added 13 patients with confirmed ZIKV infections. Prior to this report, the Sri Lankan MoH had not reported cases of Zika and no literature had been published confirming transmission in the country. A serologic study of a suburban community in Ratmalana in the Colombo district in 2017 demonstrated IgG and neutralizing antibodies to ZIKV, but neutralization testing for related viruses was not performed.^74^

#### Thailand.

Thailand maintains a strong surveillance system for ZIKV with laboratory confirmation of cases, and data are made publicly available on the MoH website.^75^ Cases are regularly detected, and evidence for ongoing transmission is also supported by cases diagnosed among travelers.^76^^,^^77^ Ministry of Health data on reported cases indicated that in 2020 there were 239 reported Zika cases, compared with 63 cases in 2021 and 190 cases in 2022.^78^ In 2023, however, the MoH reported a 3-fold increase in Zika cases that, as of December 31, reached a total of 758 cases in 36 provinces, including clusters of cases in Chanthaburi, Phetchabun, and Bangkok. Some provinces reported their first ever detected Zika cases. Overall, 33 cases were in pregnant women with laboratory-confirmed ZIKV infection, and there were 15 cases of Zika-associated microcephaly.^79^ Zika-associated developmental disorders have been reported from Thailand previously, with a total of 15 cases of CZS identified from 2016–2022.^80^ In addition, in 2022, a case of fetal microcephaly was detected on ultrasound in a pregnant woman in France who had traveled to Thailand during the first trimester of her pregnancy; sequencing of RNA from fetal brain tissue following medical termination of pregnancy was consistent with ZIKV circulating in Thailand.^81^ Genetic sequencing of ZIKV RNA obtained from patients in Thailand from 2020–2023 was consistent with ZIKVs circulating previously in Thailand, Cambodia, and Myanmar, rather than with ZIKVs sequenced from French Polynesia and the Americas within the past decade.^80^

### Western Pacific Region.

Following the initial outbreak activity in the Federated States of Micronesia in 2007, a large outbreak began in French Polynesia in 2013, and sporadic cases, small clusters, and outbreaks of Zika were reported by health ministries in various other Pacific Island countries (PICs) in subsequent years.^82^^,^^83^ Some increased reporting also occurred in larger countries in the region, likely related to improved awareness and increased testing. Though infrequent, probable and confirmed cases of ZIKV-associated microcephaly and CZS have been reported from Cambodia, French Polynesia, Lao People’s Democratic Republic, and Vietnam.^84^^–^^87^ In general, however, due to limited testing capacity and surveillance infrastructure, particularly in many small jurisdictions, information on the incidence and trends of ZIKV transmission in the Western Pacific Region remains limited. Key data on transmission in the region over the past 2 years include the following.

#### Singapore.

In April 2023, a cluster of ZIKV infections was detected in in the Kovan District of Singapore.^30^ The number of cases peaked in May and decreased thereafter, but sporadic cases continued to be reported, and as of December 31, 2023, a total of 30 cases had been detected.^88^ Because of the infrastructure and access to health and environmental services within the country and the resources available, including robust vector-borne disease surveillance and control under the National Environment Agency, it is likely that a swift and comprehensive response limited the extent of this outbreak.

#### Pacific Island countries and territories.

A review of arboviral disease outbreaks in PICs from 2014–2020, published in 2022, found that there had been 18 Zika outbreaks affecting 14 countries and territories in the subregion.^89^ Testing in the region increased during the PHEIC, particularly with awareness of the risks of congenital disease, but ceased in many countries from 2018 onwards, and confirmed case reports in the subregion have been sparse. Reports of arboviral disease cases overall are frequently delayed, incomplete, and based on syndromic surveillance without laboratory confirmation.^89^

The Solomon Islands experienced Zika outbreaks in 2015 and 2016, and a serosurvey conducted in 2018 found 56% IgG seropositivity to flaviviruses, although the proportion attributable to prior ZIKV versus DENV infection was not discernable.^89^^,^^90^ In September 2023, the MoH of the Solomon Islands reported detection of ZIKV infections upon enhanced surveillance with RT-PCR testing performed ahead of the Pacific Games, which was hosted in the Solomon Islands in November–December 2023.^91^ As of October 11, 2023, seven Zika cases had been detected and community and traveler health advisories had been issued.

In Papua New Guinea, a seroprevalence study among military personnel conducted on specimens collected in 2019 suggests that there might have been more extensive recent circulation of ZIKV in the country than previously thought.^92^ A recent case of probable Zika was also diagnosed in a U.S. traveler after a visit to Papua New Guinea; the case was reported as probable in accordance with U.S. national surveillance case definitions, but complete test results were unavailable (personal communication with the U.S. CDC).

## DISCUSSION

This review provides an update on ZIKV epidemiology as of late 2023, affirming continued transmission of ZIKV in several countries globally despite limited surveillance and highlighting evidence of autochthonous mosquito-borne transmission in countries not previously included in the WHO maps, namely Sri Lanka, Guinea, and Mali.^33^^,^^34^^,^^41^^,^^93^

The decline in reported Zika cases in high-disease-burden countries since the PHEIC appears reassuring and reflects, in large part, true reduction in transmission. However, surveillance is limited in most countries, particularly in the face of competing public health priorities, lack of public and health care worker awareness, and challenges in diagnostic test performance. The negative impact of disease notifications on tourism may further disincentivize the performance of surveillance for ZIKV and other epidemic-prone pathogens.^94^ Definitive confirmation of ZIKV autochthonous transmission remains challenging because of the short window of RNA detectability following infection, cross-reactivity and persistent detectability of IgM antibodies, and the challenges of plaque reduction neutralization tests to confirm ZIKV infection and exclude cross-reactivity from other circulating flaviviruses. Plaque reduction neutralization tests are resource and time intensive to perform, require laboratories at the appropriate biosafety level, and necessitate staff with sufficient technical expertise to interpret results. Surveillance data from the Americas, where reporting appears to be most consistent and where arboviral surveillance is conducted in all member states, has shown year-to-year consistency indicative of endemicity in some geographic locations but apparent interruption in others.^25^ Increased case numbers in Thailand in 2023 and periodic clusters of disease in India and Singapore not only are reminders of ZIKV circulation outside of the Americas but also highlight the need for strengthened surveillance to improve understanding of epidemiologic trends.

In Guinea and Sri Lanka, ZIKV transmission was detected for the first time through specific research efforts and not through routine MoH surveillance systems, which might not include ZIKV testing or might be insufficiently sensitive to detect low-level circulation. Similarly, active transmission was recognized in two countries, the Solomon Islands and Maldives, through enhanced, nonroutine testing practices or because of ZIKV diagnosed in travelers; in neither case had ongoing transmission been reported through in-country routine testing or surveillance. Although routine surveillance and testing are very important to monitor disease patterns, in the absence of such efforts, additional data sources can be helpful in improving awareness of the local transmission risk. Travel surveillance networks can provide supplementary data because returning travelers are often more likely to seek care and obtain extensive diagnostic services in their home countries; furthermore, travelers are more likely to be immunologically naïve and thus have clinically evident infections.^77^^,^^95^^–^^97^ In addition, research studies can be valuable to detect transmission given the laboratory resources often available in such studies and, in countries with *Aedes* (*Stegomyia*) vectors but without Zika surveillance, might be the only way ZIKV circulation could be recognized.

In areas with ongoing or clusters of circulation, ZIKV-associated microcephaly and CZS cases have been reported infrequently since 2017, with the recent exception of increased microcephaly reports from Thailand, and the reasons for this are not clear. Sequelae of congenital infection were identified more readily at the height of the outbreak in the Americas, when surveillance and reporting of congenital development defects were enhanced in many countries. However, surveillance of Zika-associated birth defects has diminished or ceased in many areas and, when present, is usually not linked with communicable disease surveillance systems. Health systems in low- and middle-income countries often lack capacity for birth defect surveillance, and if done, performance of basic measures such as head circumference is of variable quality.^98^^,^^99^ In the context of minimal ZIKV transmission, neurological sequelae or adverse pregnancy outcomes would occur too infrequently to generate cluster signals, and the lack of detection might be further hampered by underreporting of birth defects, particularly in resource-constrained countries.^100^ Some authors previously postulated that ZIKV-associated microcephaly and other developmental defects occurred only where there was circulation of ZIKV with a S139N mutation; however, the recent report of radiological detection of fetal congenital developmental defects in India and reports of congenital microcephaly in Thailand where circulating strains have not demonstrated this mutation suggest that it is not a prerequisite for adverse pregnancy outcomes.^71^^,^^80^^,^^101^

Vector surveillance efforts have increased awareness of the areas at risk for introduction and transmission of ZIKV because of new detections of *Ae. aegypti* and *Ae. albopictus* in areas where these vectors were not previously present/documented, including in parts of Europe.^38^^,^^102^ While findings are in part attributable to increased vector surveillance efforts, it is expected that areas with established *Aedes* mosquito populations will expand further due to vector movement by conveyances, vector adaptation to peridomestic environments, accumulation of vector breeding sites in urban settings, limitations in vector surveillance and control particularly in jurisdictions with limited resources, and climate change in areas where temperature and precipitation changes favor expansion of vector populations.^103^^,^^104^ This is particularly important in fragile settings where absence of infrastructure, crowding, and population movement are conducive to the spread of vector-borne diseases.^36^ In addition, there are concerns that, over time, increases in the size of ZIKV immunologically naïve populations in areas without ongoing transmission will lead to large-scale outbreaks because of ZIKV introduction from geographic pockets of endemicity.^105^

Zika virus thus remains a public health challenge, and transmission continues across multiple regions where infection, disease, and sequelae are likely underrecognized. The growing proportion of people living in areas with competent *Aedes* (*Stegomyia*) vectors but without prior ZIKV infection and subsequent immunity will be vulnerable to infection if ZIKV is introduced or reintroduced locally, and travelers to areas of ZIKV transmission and their sexual partners, particularly pregnant women, are also at risk of adverse outcomes.^4^ In response to the threat of ZIKV and other *Aedes*-borne arboviruses, the WHO launched the Global Arbovirus Initiative in 2022 with an emphasis on preparedness and response to arboviral epidemics. Within the proposed framework, member states are encouraged to strengthen epidemiological surveillance through support for monitoring and testing practices and implementation of clear case definitions and epidemiologically appropriate laboratory testing algorithms. Surveillance efforts for ZIKV could focus in particular on potentially high-yield patient populations such as patients with fever and rash, neonates with birth defects including microcephaly, and pregnant women with defined adverse pregnancy outcomes.^106^ Clinicians should be equipped through continuing education programs in clinical diagnosis and management, vector surveillance and control activities should be strengthened, and health authorities should engage communities using validated risk communication tools to enable symptom recognition and protective measures against ZIKV infection. Research and development of improved diagnostics and medical countermeasures are ongoing priorities to ensure better diagnosis and early detection of ZIKV transmission.
